# A machine learning approach identifies 5-ASA and ulcerative colitis as being linked with higher COVID-19 mortality in patients with IBD

**DOI:** 10.1038/s41598-021-95919-2

**Published:** 2021-08-13

**Authors:** Satyaki Roy, Shehzad Z. Sheikh, Terrence S. Furey

**Affiliations:** 1 Department of Genetics, University of North Carolina, Chapel Hill, USA; 2 Departments of Medicine and Genetics, Center for Gastrointestinal Biology and Disease, University of North Carolina, Chapel Hill, USA; 3 Departments of Genetics and Biology, Center for Gastrointestinal Biology and Disease, University of North Carolina, Chapel Hill, USA

**Keywords:** Crohn's disease, Ulcerative colitis, Computer science

## Abstract

Inflammatory bowel diseases (IBD), namely Crohn’s disease (CD) and ulcerative colitis (UC) are chronic inflammation within the gastrointestinal tract. IBD patient conditions and treatments, such as with immunosuppressants, may result in a higher risk of viral and bacterial infection and more severe outcomes of infections. The effect of the clinical and demographic factors on the prognosis of COVID-19 among IBD patients is still a significant area of investigation. The lack of available data on a large set of COVID-19 infected IBD patients has hindered progress. To circumvent this lack of large patient data, we present a random sampling approach to generate clinical COVID-19 outcomes (outpatient management, hospitalized and recovered, and hospitalized and deceased) on 20,000 IBD patients modeled on reported summary statistics obtained from the Surveillance Epidemiology of Coronavirus Under Research Exclusion (SECURE-IBD), an international database to monitor and report on outcomes of COVID-19 occurring in IBD patients. We apply machine learning approaches to perform a comprehensive analysis of the primary and secondary covariates to predict COVID-19 outcome in IBD patients. Our analysis reveals that age, medication usage and the number of comorbidities are the primary covariates, while IBD severity, smoking history, gender and IBD subtype (CD or UC) are key secondary features. In particular, elderly male patients with ulcerative colitis, several preexisting conditions, and who smoke comprise a highly vulnerable IBD population. Moreover, treatment with 5-ASAs (sulfasalazine/mesalamine) shows a high association with COVID-19/IBD mortality. Supervised machine learning that considers age, number of comorbidities and medication usage can predict COVID-19/IBD outcomes with approximately 70% accuracy. We explore the challenge of drawing demographic inferences from existing COVID-19/IBD data. Overall, there are fewer IBD case reports from US states with poor health ranking hindering these analyses. Generation of patient characteristics based on known summary statistics allows for increased power to detect IBD factors leading to variable COVID-19 outcomes. There is under-reporting of COVID-19 in IBD patients from US states with poor health ranking, underpinning the perils of using the repository to derive demographic information.

## Introduction

Coronavirus Infectious Disease 2019 (COVID-19) is a respiratory illness caused by severe acute respiratory syndrome coronavirus 2 (SARS-CoV-2). Since its inception in 2019, COVID-19 has claimed 2.24 million lives by early February 2021^1^. Manifestations of COVID-19 range from asymptomatic carriers to fulminant disease characterized by sepsis and acute respiratory failure. Individuals with preexisting conditions such as diabetes, obesity, lung disease or cardiovascular conditions are at high risk of succumbing to COVID-19. However, the interrelationship between COVID-19 mortality and autoimmune disorders is still being studied closely^2^. Inflammatory bowel disease (IBD) is characterized by chronic inflammation of the gastrointestinal tract that is generally believed to be largely caused by an aberrant response to the enteric microbiota by the host immune system. Corticosteroids, immunomodulators, and biologics that are used to treat IBD may increase patient susceptibility to viral infection while also conversely dampening the immune response^3^. Yu et al. recorded the condition of COVID-19 in 102 IBD patients in the form of an online questionnaire to guide lifestyle management of IBD patients^4^. However, these studies are hindered by the lack of substantial available data on IBD patient outcomes after the contraction of COVID-19.

Several studies have attempted to better understand the effect of IBD on COVID-19 susceptibility and progression. Attauabi et al. used binary linear and logistic regression models to show that the patients of immune-mediated inflammatory diseases (IMID), like IBD, were less susceptible to COVID-19 than patients without IMID^5^. Similarly, Gutin et al. presented case studies to show that there is little evidence to suggest that patients with IBD are at increased risk of acquiring COVID-19^6^. Al-Ani et al. carried out a literature review to prescribe that IBD patients should desist from immune-suppressing medications until infection resolution^7^. Aysha et al. reported that a sub‐group of IBD patients with mild COVID-19 disease initially presented with diarrhoea rather than respiratory symptoms, leading to delayed diagnosis^8^, while D’Amico also reported that diarrhoea is a common symptom of COVID-19 in IBD patients^9^. Neurath et al. posed a question on the role of immunosuppression and immunomodulation on the COVID-19 outcome of IBD patients^10^. While identifying key challenges and recommendations for IBD patients in COVID-19, Dotan et al. suggested that immune-modifying medication including biological therapy renders IBD patients more susceptible to infection^11^. Laurie et al. showed that telemedication-based treatment can help address the racial, socioeconomic and demographic disparities in medical care for IBD patients with COVID-19^12^. The Gastroenterological Society of Australia recommended that the IBD patients should continue minimum levels of immunosuppression as more information is gathered on the effective control measures^13^. An analysis of clinical data from a cohort of Italian IBD patients showed that the active IBD, old age and comorbidities were associated with more fatal outcomes^14^. It is worth noting that these analyses were carried on a limited amount of data from IBD patients with COVID-19 symptoms, potentially presenting findings that have low statistical significance.

The IBD community is seeking answers to two core questions: (1) are IBD patients more vulnerable to COVID-19 related complications and mortality? (2) Should treatment guidelines for IBD patients be modified in the COVID era? Data sparsity is a major challenge in the way of developing effective recommendations^15^. To overcome this challenge, we employ a sampling approach that generates patient data from existing trends in COVID-19 outcomes reported in IBD patients. Sampling approaches have been used to generate large volumes of data from a small initial dataset, under the assumption that the latter is a true representation of the actual population^16^. Existing sampling techniques, employing probability distributions^17^, Bayesian networks with latent variables^18^ and deep learning^19^, ensure considerable variation in the initial dataset to avoid biased inferences in the generated data.

The sampling approach presented in this work uses a stratified, multistage random sampling technique^20^. The stratification is done based on preassigned clinical features of patients, ensuring the sampled data preserves the likelihood of occurrence of the features given the outcome reported in the original data. We used supervised and unsupervised machine learning-based statistical methods to model large amounts of IBD patient data. Using these data, we present a comprehensive study of the effects of features such as gender, age, medication usage, IBD subtype (CD or UC), disease severity and demographics of IBD patients, and the accuracy of using them to predict COVID-19 outcomes. Our analysis captures both primary and secondary factors contributing to mortality in Crohn’s disease as well as ulcerative colitis patients due to COVID-19. Secondary factors are clinical characteristics that help distinguish the outcomes of any two patients if their primary factors are highly similar. Finally, we explore the challenges of inferring demographics from the existing COVID-19/IBD dataset.

## Methods

### Dataset

#### COVID-19/IBD repository

We acquired data from IBD patients who tested positive for COVID-19 from the Surveillance Epidemiology of Coronavirus Under Research Exclusion Inflammatory Bowel Disease (SECURE-IBD)^21^ repository jointly created by researchers of University of North Carolina, Chapel Hill and Mount Sinai, NY, USA. This international, pediatric and adult database based on collaborative participation (1) monitors and reports on outcomes of COVID-19 occurring in IBD patients and (2) provides the IBD community with updates on affected numbers based on demography. The repository is populated with data that is compiled within the UNC REDCap (Research Electronic Data Capture) system, a secure, web-based electronic data capture tool hosted at the University of North Carolina at Chapel Hill. Disease activity (mild, moderate, remission or unknown) was determined based on the assessment of a physician during a COVID-19 related emergency room visit or upon hospitalization. These data include several patient characteristics, namely, age, sex, smoking status, medication usage, severity of IBD, number of preexisting conditions (comorbidities), along with COVD-19 outcomes, namely whether the patient was an outpatient, hospitalized, admitted to the ICU, ventilated and/or deceased. Data is based on country-wise and US state-wise counts of voluntarily reported COVID-19 cases in IBD patients.

#### American health rankings

We also acquired data from a comprehensive repository of US national health statistics provided on a state-to-state basis^22^. Scores for a multitude of health-related features are provided that are based on a history of environmental and socioeconomic data. The repository offers an annual report with a variety of features, where the major classes include behaviors, community environment, policy, clinical care and outcomes with a plethora of sub-categories (see health ranking sub-criteria in the “Results” section). Each sub-category is associated with a list of state names ranked on calculated scores in the order of healthy to unhealthy.

#### Preprocessing and dataset generation

Using data in the SECURE-IBD repository (2.1.1), we generated a table for each feature (age, sex, smoking status, medication usage, severity of IBD, and the number of comorbidities) that summarizes the percentage of the COVID-19/IBD population that fall into the following outcomes: outpatients (OP), hospitalized and recovered (H-R), and hospitalized resulting in death (H-D) (see Table 1). We propose a random sampling approach using these feature tables to generate a complete patient dataset for any number of desired patients with the following set of characteristics: Patient ID, Gender, Age, Medication Usage, Number of Comorbidities, Smoking Status, IBD subtype, IBD Severity, Country, State, Hospitalization Status.

**Table 1:** We subdivided patients into nine age groups and calculated the proportion of patients within each subgroup that were outpatients (OP), hospitalized resulting in death (H–D) and hospitalized and recovered (H–R) outcomes.

	Total	OP (%)	H–D (%)	H–R (%)
0–9 years	17	88	0	12
10–19 years	296	94	0	6
20–29 years	649	90	0	10
30–39 years	634	85	0	15
40–49 years	508	79	0	21
50–59 years	470	70	2	28
60–69 years	274	55	7	38
70–79 years	126	47	10	43
$\ge$ 80 years	84	45	24	31

For each simulated patient ***p***, we generated these features and outcomes as follows:

1. Determine the patient gender (${f}_{i}$ = male or female) based on the rule:
  1 $${pr(f}_{i})=\frac{n\left({f}_{i}\right)}{{\sum }_{j}n({f}_{j})}.$$
  Here $n\left({f}_{i}\right)$ is the number of people with gender *f*_*i*_. Given N = ${\sum }_{j}n({f}_{j})$, the number of male and female patients follow the multinomial distribution given by:$\frac{N!}{{\prod }_{{f}_{i}}n({f}_{i})} \times {\prod }_{{f}_{i}}pr{\left({f}_{i}\right)}^{n\left({f}_{i}\right)}$. Therefore, the expected number of patients of any given feature value *f*_*i*_ is calculated as $n\left({f}_{i}\right)=N \times p\left({f}_{i}\right)$.
2. Given the hospitalization status *s*, we now calculate all other features, one at a time, based on the conditional probability of a feature given status *p (f | s)*.
3. *Observations* We assume that gender is conditionally independent of other features *f*, i.e., *pr (f |gender)* = *pr(f)*. However, this approach preserves the probabilities between input features and outcomes as summarized in the SECURE-IBD summary tables, since the likelihood of observing a feature *f* in the generated dataset is given by
  2 $${\sum }_{{o}_{j}}pr\left({o}_{j}\right) \times pr\left(f|{o}_{j}\right)= {\sum }_{{o}_{j}}pr\left(f, {o}_{j}\right)=pr\left(f\right).$$

It is noteworthy that Step 2 of the random sampling approach ensures that the simulated patient dataset preserves the likelihood of each feature value given the observed variable (i.e., hospitalization status) from the original dataset. By summing up the marginal probabilities in Eq. (2), we show that the likelihood of finding a feature value in the generated dataset corresponds to the probabilities defined in the SECURE-IBD summary tables.

### Supervised learning methods

Supervised machine learning (ML) algorithms learn a function that maps the input training data (i.e., features) to some output labels^23^. In this work, we consider the following supervised learning techniques, evaluating each using cross-fold validation (see^24–34^ for more details on these ML approaches).

- *Support vector machine* (SVM) is used for classification and regression problems that map sample data to high-dimensional feature spaces. SVM operates on hyperplanes—decision boundaries in high-dimensional space—that define the class for the data points. The objective of SVM is to maximize the separation between the training data points and the learned hyperplane, and later use this separation to classify new samples. SVM is memory efficient and effective for datasets with few samples^24,25^.
- *Stochastic gradient descent* (SGD) is an iterative strategy that fits the training data to an objective function that is used to classify new samples^26,27^. SGD is a stochastic variant of the popular gradient descent (GD) optimization model^27,28^. In GD, the optimizer starts at a random point in the search space and reaches the lowest point of the function by traversing along the slope. Unlike GD that requires calculating the partial derivative for each feature at each data point, SGD achieves computational efficiency by estimating derivatives on randomly chosen data points.
- *Nearest centroid* (NC) is a classification approach that represents each learned class from the training data by the centroid of its members. Subsequently, it assigns each new sample data point to the cluster whose centroid is the closest. NC is particularly effective for non-convex classes and does not suffer from any additional dependencies on model parameters^29^.
- *Decision trees* (DTs) are a classification and regression technique that assigns target labels based on decision rules inferred from data features of the training samples^30,31^. DT maintains the decision rules using a tree. A new data point is repeatedly assessed using the conditional statement at a particular tree node and branches off to a new node based on this conditional until a leaf node is reached. The new data point is then assigned the class of the leaf node.
- *Gaussian Naive Bayes* (NB) are a class of fast, probabilistic learning techniques that apply the Bayes’ theorem to assign labels to the new data points^32^.
- *Supervised neural network* (SNN) or multilayer perceptron (MLP) is a deep artificial neural network comprising several neural network units, called perceptrons. Each perceptron is a function that combines any input with learned weights (or hyperparameters) to generate an output value. MLP consists of an input layer that receives the input data, a set of hidden layers serving as computational engines and an output layer that makes a prediction based on the input. MLP training operates in two stages: a forward pass and a backward pass. The forward pass propagates the signal from the training input to the output layer, measuring the output prediction against the ground truth. The backward pass pushes data from the known output towards the input layer, modulating the hyperparameters to enable the prediction to best fit the ground truth of the training data^33^.

Note that supervised ML approaches generally yield reliable prediction accuracy. However, they often suffer from overfitting or convergence issues^34^. Each of the above approaches has its advantages and disadvantages. For instance, SVM works well when the underlying distribution of the data is not known. However, it is prone to overfitting when the number of features is much greater than the number of samples. SGD converges quickly for large datasets, but it is restrictive because it may require fitting a large number of hyper-parameters. Conversely, DT involves almost no hyper-parameters but often entails slightly higher training time. Unlike DT, NB requires less training time but works on the intrinsic assumption that all the attributes are mutually independent. Finally, NC is a fast method but is not robust to outliers or missing data. In the context of our work, we try out several features to get a broad sense of the best features that are applicable in most scenarios. The supervised and unsupervised ML approaches were implemented using the Python Scikit-learn library^34^.

### Metrics

We use the following metrics to evaluate each of the classifiers and to determine the importance of sample features:

- *Accuracy* function measures the fraction of matches between the predicted and actual labels in a multi-label classification, i.e., the ratio of correctly predicted observations to the total observations. It can be calculated as:
  $$ACC=\frac{TP+TN}{TP+TN+FP+FN}.$$
- In the above equation, TP, TN, FP, FN denote true positive, true negative, false positive and false negative, respectively. Other metrics of accuracy are *precision (pr)*, *recall (re)* and *F-score (fs)*, measured as:
  $$pr=\frac{TP}{TP+ FP} re=\frac{TP}{TP+ FN} fs=2 \times \frac{pr \times re}{pr+re}.$$
- *Feature importance* is calculated by employing the extra trees classifier estimator that fits randomized decision trees (called extra-trees) on data samples^35^. The memory and computation overhead of this approach can be controlled by regulating the size of the extra trees. The nodes in the tree are split into sub-trees resulting in high accuracy (i.e., drop in impurity). Thus, feature importance is measured as the total reduction in impurity affected by that feature^35^.
- *Multiple linear regression* (MR) is a statistical approach that measures the linear relationship between the independent and the dependent variables *x* and *y* of a function *y* = *g(x)*. MR generates this linear relationship $\widehat{y} = {\beta }_{0}+ {\beta }_{1}{x}_{1}+ {\beta }_{2}{x}_{2}+\dots +\epsilon$, where ${\beta }_{i}$ is the coefficient that captures the contribution of feature ${f}_{i}$ towards the dependent variable *y*, while β_0_ and $\epsilon$ are the intercept and error terms, respectively^36^. In the context of this work, the independent variables are the gender, age group, medication usage, number of comorbidities, smoking status, IBD subtype, and IBD severity, while the dependent variable is the outcome (namely, outpatient, hospitalized/recovered and hospitalized/deceased).
- *Multinomial logistic regression* (MLR) is also a statistical tool that fits the data to a line to find the association between the independent and dependent variables. Unlike MR, the data is passed through a logistic function that predicts the target or dependent variable. Moreover, the dependent variable in MR is continuous, while in MLR it is categorical, i.e., assuming a limited number of possible discrete values.

### Statistical operations

Given any pair of vectors $v$ and $\widehat{v}$, we perform these statistical performance measures:

- *Mean squared error* (MSE) is calculated as ${\frac{1}{n}{\sum }_{i}({\widehat{v}}_{i}- {v}_{i})}^{2}.$
- *Pearson Correlation Coefficient* (PCC) between $v$ and $\widehat{v}$ measures the strength of a linear association between two variables, where the value PCC = 1 is a perfect positive correlation and − 1 is perfect negative correlation.
- Principal component analysis (PCA) is a dimension reduction approach that projects each data point onto the first few principal components to achieve a lower dimensional representation of the data, while preserving most of the variation in the data. We consider the first two principal components, which capture the highest variance in the data^37^. Therefore, features contributing the most towards the first and second principal components are called *primary* and *secondary* factors, respectively.
- *Cosine similarity* is the similarity between two vectors $v$ and $\widehat{v}$ on a scale from 0 and 1, calculated as the cosine angle between them, i.e., $cos\left(v,\widehat{v}\right)= \frac{\widehat{v} v}{\left|\left|v\right|\right|\times \left|\left|\widehat{v}\right|\right|}$. We apply it to measure the extent of co-occurrence of an input feature *f* and output *o*. To understand this, we define indicator variable ${{\varvec{I}}}_{{\varvec{c}}}$ such that ${{\varvec{I}}}_{{\varvec{c}}}=1$ if any condition *c* (say, age greater than 40) holds, and 0 otherwise. Let each patients *p* in the dataset of |P| patients have a feature *f* value *f(p)* and outcome *o(p)*, respectively. For each categorical value of feature *f* (${f}_{i}$, where i = 0, 1, 2,…) and outcome *o* (${o}_{j}$, where j = 0, 1, 2,…), we generate two vectors ${v}_{f}=\left\{{1}_{c}\right|c:f(p)={f}_{i} \forall p\in P\}$ and ${v}_{o}=\left\{{1}_{c}\right|c:o(p)={o}_{j} \forall p\in P\}$. Then $cos\left(v,\widehat{v}\right)$ informs how strongly feature value ${f}_{i}$ aligns with outcome ${o}_{j}.$
  For instance, let there be *|P|*= *5* patients with following medications: Sulfasalazine/ mesalamine, Budesonide, Oral/parenteral steroids, 6MP/azathioprine monotherapy, Sulfasalazine/mesalamine and following outcomes: outpatients, hospitalized recovered, and hospitalized deceased. Then, ${v}_{med=\mathrm{Budesonide} }=\{$ 0,1,0,0,0} and ${v}_{out=\mathrm{death} }=\{$ 0,0,0,0,1} and $\mathrm{cos}\left({v}_{med=Budesonide }, {v}_{med=Budesonide}\right)$ = 0 suggests no association (by co-occurrence) between feature medication equals Budesonide and outcome equals death.
- *Kendall’s tau* is a measure of the correlation for ordinal data. Values close to 1 indicate strong agreement in rank order, while − 1 indicate strong disagreement^38^.
- *Z-score* is the number of standard deviations by which a data point is above or below the mean value. For any data point *x* is calculated as:
  $$z=\frac{x-\mu }{\sigma },$$
- *One sample proportion Z-test* is a standard hypothesis testing approach. Given the number of trials and successor trials, one can test a null hypothesis (H_0_) whether the proportion (i.e., fraction of successful trials) of the data equals a prespecified value.

### Ethics approval

As stated on the SECURE-IBD website, the created registry “contains only de-identified data, in accordance with *HIPAA Safe Harbor De-Identification standards*. The UNC-Chapel Hill Office for Human Research Ethics has determined that storage and analysis of de-identified data does not constitute human subjects research as defined under federal regulations [45 CFR 46.102 and 21 CFR 56.102] and does not require IRB approval” (see https://covidibd.org/faq/).

## Results

### Analytical design of the study

**Figure 1:**
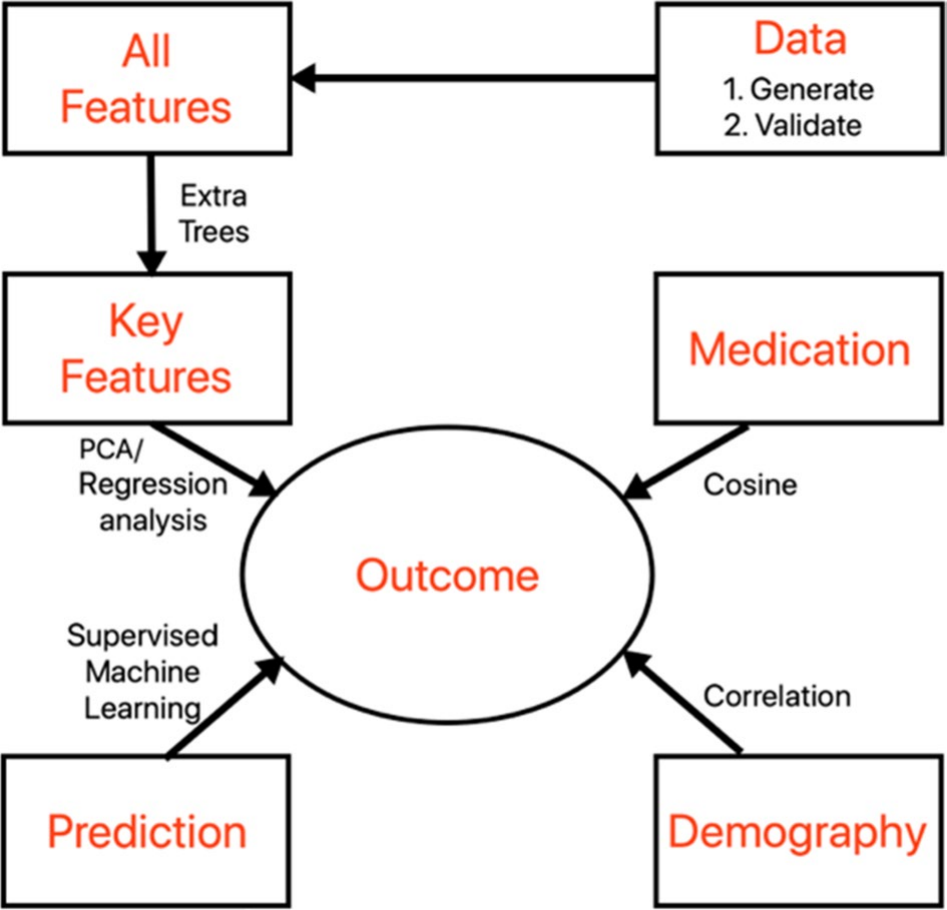
Outline of salient contributions.

We outline the experimental design of our work in Fig. 1. First, we apply a random sampling approach on the summary statistics (introduced in “Preprocessing and dataset generation” section) of COVID-19 outcomes of IBD patients (outpatients, hospitalized/recovered and hospitalized/deceased) to generate a large-scale clinical dataset. We then validate the dataset by comparing the trends in the odds ratio of logistic regression analysis of the generated dataset against that of the real data (as reported in the Brenner et al.^39^). Next, we apply randomized decision trees (i.e., extra trees classifier) to identify the subset of key features contributing to the outcome.

We perform the following analyses: (1) use regression analysis to quantify the contribution of the features towards the outcome and principal component analysis to identify the interrelationship between the primary and second features on the patient COVID-19 outcomes; (2) apply supervised and unsupervised machine learning approaches to estimate the accuracy of the significant features in predicting the outcomes; (3) calculate cosine similarity to determine the association between use of each medication and subsequent outcome, and (4) perform correlation studies on American Health Rankings (discussed in “American health rankings” section) to reveal the perils of predicting the locations with the highest concentration of COVID-19/IBD patients in the United States. We discuss the implications behind our findings in light of other reported studies on COVID-19/IBD patients.

### Generation of patient data

We first applied our random sampling approach (“Preprocessing and dataset generation” section) to generate a COVID-19/IBD clinical dataset for 20,000 patients (Table 2) based on the summary statistics of 1739 Crohn’s disease and 1323 UC patients reported by Brenner et al.^39^. In their study, they performed multivariable logistic regression to calculate the effects of age, sex, IBD subtype (CD vs UC/IBD-U), disease activity, smoking status, body mass index ≥ 30, and the number of comorbidities (0, 1, ≥ 2) on the primary outcome of severe COVID-19, defined as a composite of ICU admission, ventilator use, and/or death. They incorporated tumor necrosis factor (TNF) antagonist use (versus not) and sulfasalazine/5-aminosalicylate (5-ASA) use (vs not), as these were the two most commonly reported medication classes, and systemic corticosteroid use (vs not) on the basis of increased risk of infectious complications. A secondary outcome was the composite of any hospitalization and/or death. Finally, they calculated adjusted odds ratios (aOR) and 95% confidence intervals (CI) for (1) ICU/Vent/Death, (2) Hospitalization or Death and (3) Death, for each demographic or disease characteristic. In order to validate our proposed random sampling approach, we evaluated whether the relative order of the features ranked in the decreasing order of odds ratio that we similarly calculated based on our generated dataset matches the ordering based on the original data using Kendall’s tau score. We ordered the input features in the generated dataset in the decreasing order of odds ratio for two outcome scenarios, namely (a) ICU/Ventilation/Death and (b) Death (see Supplementary Fig. S1). The ordered odds ratio on the generated dataset and real data shows Kendall’s tau score (on a scale of − 1 to + 1) of (a) 0.73 and (b) 0.55, respectively, against that of the reported COVID-19/IBD data. This shows that the data we generated through random sampling preserves the trends of the original COVID-19/IBD data.

**Table 2:** 5 rows of the dataset showing the features and outcomes for a patient with the features medication usage (medication), number of comorbidities (comorbidity), smoking status (smoking), IBD subtype (condition) and IBD severity (severity).

Patient ID	Gender	Age group	Medication	Comorbidity	Smoking	Condition	Severity	Country	State
0	Male	20–29	Anti-TNF	0	Non-smoker	UC	Remission	United States	New York
1	Male	20–29	Anti-TNF	2	Non-smoker	UC	Remission	Germany	–
2	Male	50–59	IL 12/23	0	Non-smoker	Crohn	Mild	United States	New York
3	Female	30–39	Anti-TNF	1	Non-smoker	UC	Remission	United States	New York
4	Male	40–49	Sulfasalazine	1	Non-smoker	UC	Remission	Spain	–

### Age, medication usage, number of comorbidities and IBD severity are the key features affecting COVID-19 outcomes

**Figure 2:**
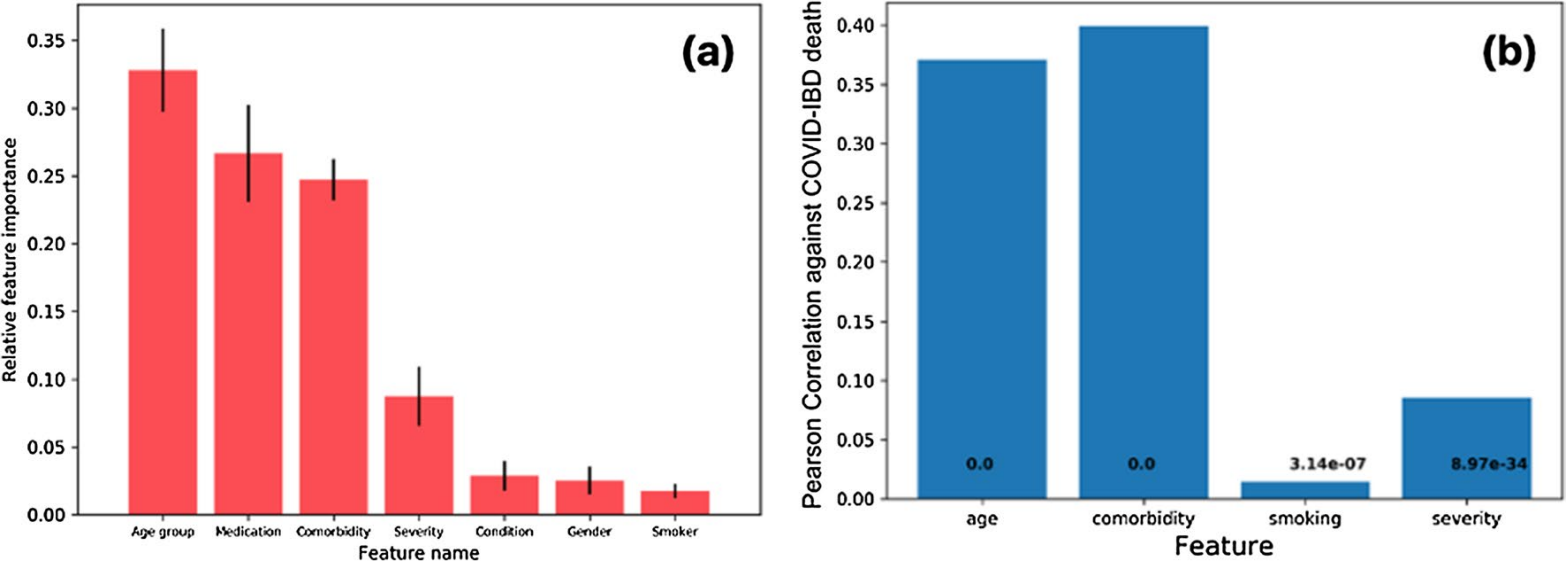
Identification of discriminatory features. (**a**) the importance of features based on extra trees classifier, (**b**) Pearson correlation coefficient between ranked features, namely, age, comorbidity (number of comorbidities), smoking (smoking status) and severity (IBD severity) against COVID-19/IBD death counts.

We created ranked subgroups based on quantitative ranges of the features age group, number of comorbidities, smoking status and IBD severity (Table 3). Using feature importance and multiple linear regression analysis (see “Metrics” section), we sought to identify the patient features that help discriminate among the three COVID-19 disease outcomes: outpatients, hospitalized-recovered, and hospitalized-deceased. We found that age group, medication usage and number of comorbidities have the highest importance (also refer to “Metrics” section for details on feature importance), while IBD subtype (CD or UC), smoking status and gender have the least independent feature importance (Fig. 2a).

**Table 3:** Ordinal score for feature value groups for features age, comorbidity (number of comorbidities), smoking (smoking status), IBD severity (severity).

Feature	Group	Rank
Age	0–9, 10–19, 20–29, 30–39, 40–49, 50–59, 60–69, 70–79, $\ge 80$	1, 2, …, 9
Comorbidity	0, 1, 2, 3 +	0, 1, 2, 3
Smoking	Non-smoker, smoker	0, 1
Severity	Mild, moderate, severity	0, 1, 2

We found that age and number of comorbidities are correlated with the number of patients who died (Pearson’s correlation coefficient > 0.35), while smoking status and IBD severity are largely uncorrelated (Fig. 2b). Interestingly, age is contingent on a variety of factors such as genetic background and its cumulative effect of generic responses to biological stress and environmental exposures, and there is evidence to suggest that the number of comorbidities increases with age and is larger in individuals 65 years and older^40^. Given the high potential of mutual information between age and number of comorbidities, we studied two feature combinations with either age or the number of comorbidities, specifically (a) medication usage, IBD subtype, IBD severity and age and (b) medication usage, IBD subtype, IBD severity and number of comorbidities, against outcomes (outpatients, hospitalized/recovered and hospitalized/deceased). Multiple linear regression analysis shows that age and number of comorbidities have the highest coefficients in their respective groups, followed by IBD severity and IBD subtype. This indicates that age and number of comorbidities are the key features affecting COVID-19/IBD outcomes (refer to Supplementary Tables S1, S2).

**Figure 3:**
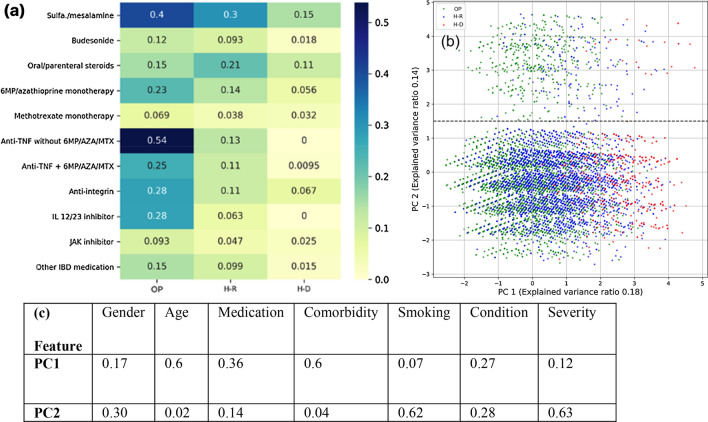
Identification of discriminatory features. (**a**) cosine similarity of 11 medications and outcome vectors, (**b**) PCA on the 7 input features labeled by three patient outcomes, (**c**) contributing weights of 7 features along PC1 and PC2.

Next, we analyzed the interrelationship between the COVID-19/IBD outcome and medication usage. To this end, we considered our generated dataset of *|P|*= 20,000 COVID-19/IBD patients, 11 IBD medications (see Fig. 3a) along with the three COVID-19 disease outcomes. For each medication *m* and disease outcome *d*, we calculated two vectors ${v}_{d}=\left\{{1}_{outcome\left(p\right)={\varvec{d}} }\right|p\in P\}$ and ${v}_{m}=\left\{{1}_{medication\left(p\right)={\varvec{m}} }\right|p\in P\}$, where the indicator variable ${1}_{{\varvec{c}}}=1$ if condition *c* is true, and 0 otherwise. Essentially, this vector represents for each patient, the combination of medications being used. We applied the cosine similarity $1-\mathrm{cos}({v}_{m}, {v}_{d})$ (see “Statistical operations” section) to compare the profiles between each medication and the outcome vector. Figure 3a shows that while using anti-TNF without immunomodulators (namely 6MP/AZA/MTX, used to treat Crohn’s disease^41^) exhibits a high similarity with the outpatient outcome, 5-ASAs (sulfasalazine/mesalamine) consistently align well with all three outcomes by exhibiting one of the highest similarities (0.4, 0.3 and 0.15) with OP, H–R and H–D among the list of administered medications. This suggests that 5-ASA usage has the highest degree of overlap with all the outcomes among all medications (see “Statistical operations” section for the details).

**Figure 4:**
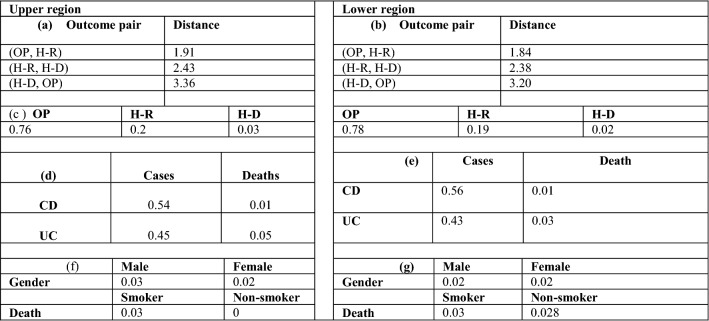
Outcomes of the PCA for the upper region (UP) and lower region (DOWN). (**a,b**) The pairwise mean Euclidean distance across all pairs of points belonging to different outcomes, (**c**) proportion of each outcome; proportion of COVID-IBD cases and deaths in (**d**) upper and (**e**) lower regions; (males, females) and (smokers, non-smokers) in (**f**) upper and (**g**) lower regions.

### IBD severity, smoking status, gender and IBD subtype are secondary factors affecting outcome

We apply principal components analysis (PCA; “Statistical operations” section) on the 7 input features of patient data and identified two components, PC1 and PC2, that account for 18% and 14% of the variance in the data (see Fig. 3b). It is noticeable that PC1 clearly demarcates the OP and H–D patients. Figure 3c once again shows that the *primary factors*, namely, age, medication usage, and the number of comorbidities, have the highest contributing weights along PC1; conversely, IBD severity, smoking status, gender and IBD subtype are strong contributors along PC2, making them key *secondary factors*—clinical characteristics that help distinguish the outcomes of any two patients if their primary factors are identical. When looking more closely at medication usage, we see that 5-ASA (sulfasalazine/mesalamine) usage is associated with most of the COVID-19/IBD deaths—approximately 47% and 68% deaths in two sample groups (defined hereafter). This lends credence to the high cosine similarity between sulfasalazine/mesalamine and H–D in Fig. 3a. Also, UC accounts for nearly three times the death than that of Crohn’s disease (3.7% and 1.3%, respectively.).

As discussed earlier in Fig. 2b, we consider three outcomes, outpatients (OP), hospitalized/recovered (H-R) and hospitalized/deceased (H–D). We noted that there is a more prominent separation between samples along PC2 at about 1.5 (dashed line in Fig. 3b). In the subsequent discussion, we term the samples above and below PC2 = 1.5 as the upper group (~ 3.5% of total patients) and lower sample group (~ 96.5% of total patients), respectively. We calculated the pairwise mean Euclidean distance across all pairs of samples such that *outcome (*${p}_{1}$*) *$\ne$* outcome (*${p}_{2}$*)* to gauge the relative difference between samples corresponding to an outcome on the PCA plot. Figure 4a,b show that for both upper and lower PC2 sample groups, samples with outcomes OP and H-R are the closest to each other, indicating that the largest variation across features is in samples corresponding to deceased patients (H–D).

Given this high variation in the H–D samples, we further analyzed the proportions of each outcome in the upper and lower groups. We found that in samples from the upper group, despite having fewer samples, there is a higher proportion (~ 3% of cases in the upper group) of H–D outcomes (Fig. 4c). A greater proportion of COVID-19/IBD cases corresponded to Crohn’s disease in both upper and lower regions (Fig. 4d), while UC patients, despite encompassing in fewer cases overall, account for a higher proportion of deaths, about 3% and 5% of total cases in the upper and lower groups, respectively (Fig. 4e). We performed statistical t-tests and found that UC patients appear more vulnerable to COVID than Crohn’s patients (Supplementary Fig. S2). Given that gender, IBD subtype and smoking are key secondary features contributing to IBD deaths, as highlighted along PC2 (Fig. 3c), the two clusters (upper and lower regions, Fig. 4) suggest a high proportion of IBD patients who are male and are smokers will have an outcome that results in death (Fig. 4f,g). Note that all smokers are in the upper region.

### Supervised learning on age, medication usage and number of comorbidities predict outcomes

**Figure 5:**
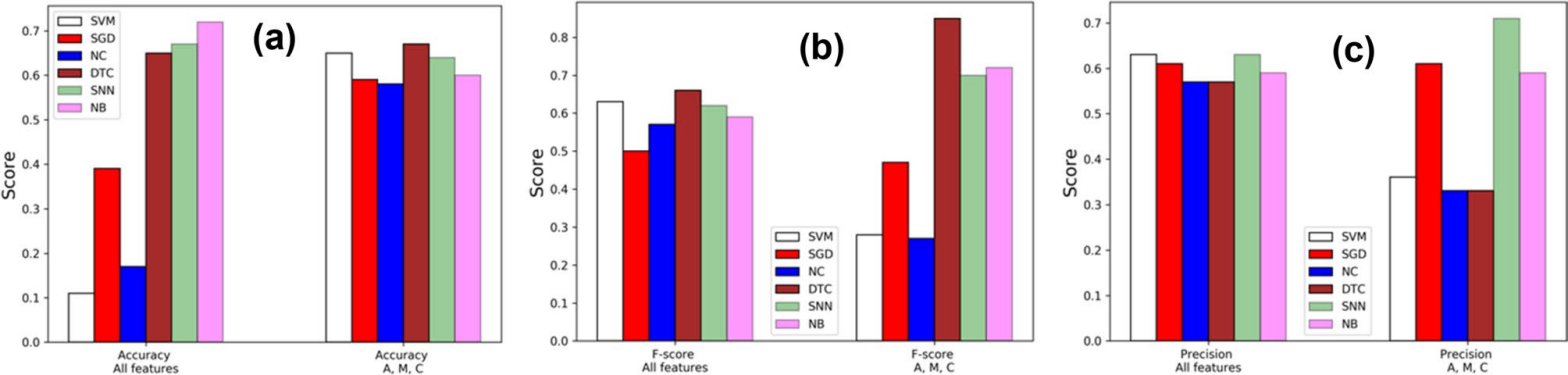
(**a**) Accuracy, (**b**) precision and (**c**) F-score in prediction of outcome using the supervised machine learning support vector machine (SVM), stochastic gradient descent (SGD), nearest centroid (NC), decision tree classifier (DTC), neural network (SN), naïve Bayes (NB).

To further evaluate the relationships between IBD features and COVID-19 outcomes suggested by the PCA, we performed classification experiments using multiple machine-learning methods (Fig. 5, Supplementary Table S3). We estimated the accuracy, precision and F-score (defined in “Metrics” section) of predicting COVID-19/IBD outcome using support vector machine (SVM), stochastic gradient descent (SGD), nearest centroid (NC), decision tree classifier (DTC), supervised neural network (SNN) and Naive Bayes (NB) classifiers. In Table 4, we summarize the hyperparameters employed in the supervised learning models. Given that the hospitalized-recovered (H-R) and hospitalized-death (H–D) account for a small fraction of the COVID-IBD dataset, we applied Synthetic Minority Oversampling^42^ to augment the input dataset to give enough training data for each outcome. We observe that the supervised machine learning classifiers with the combined feature set of age, number of comorbidities and medication usage significantly outperforms the mean accuracy of the individuals’ features.

**Table 4:** Parameters used in the supervised machine learning. (Refer to^34^ for details on these parameters).

Machine learning approach	Parameter
SVM	*Kernel*: ’RBF’; *regularization*: 1.0; *kernel function degree*: 3
SGD	*Loss*: ’hinge’; *penalty*: l2; *regularization*(α): 0.0001
NC	*Distance metric*: ’Euclidean’
DT	*Split criterion*: ’gini’; *split strategy*: ’best’; *maximum tree* depth(maxdepth): ’None
NB	*Largest feature variance*: 10 − 9

Figure 5a shows that overall, the majority of the accuracy scores for classifiers using just the three primary features exceed those classifiers that include all features. There is less deviation in precision and f-scores across approaches when using all features. The (1) precision from DTC, SNN, NB (Fig. 5b) and (2) the f-scores from SGD, SNN, NB (Fig. 5c) for classifiers using the three primary features outperform those using all features. This suggests that the predictions based on primary features exhibit a more consistent overall accuracy in outcome prediction. However, considering all the features during outcome prediction may yield an improved true positive rate, as manifested in the steady precision and f-scores across the different ML approaches.

### COVID-19 numbers in IBD patients do not align with state health indices

SECURE-IBD database provides the number of COVID cases in IBD patients reported in each state. We intended to investigate whether the frequency of cases correlated with the healthiness of each state. Using state health indices for nearly 50 criteria (Table 5), we calculated the Pearson correlation of the ranked list of states based on each state health index with the ranked list of states based on the increasing number of COVID-19 in IBD patients scaled by the population of the state. A high correlation would imply that healthy states have fewer COVID-IBD cases, and vice versa.

**Table 5:** The health ranking criteria and their sub-categories for US states.

Behaviors	Community and environment	Policy
Drug deaths	Air pollution	Immunizations—adolescent
Excessive drinking	Children in poverty	HPV Immunization females
High School graduation	Infectious disease	HPV Immunization males
Obesity	Chlamydia	Meningococcal Immunization
Physical inactivity	Pertussis	Tdap immunization
Smoking	Salmonella	Immunizations—children
	Occupational fatalities	Public health funding
	Violent crimes	

Clinical care		Outcomes
Dentists		Cancer deaths
Low birthweight		Cardiovascular deaths
Mental health providers		Diabetes
Preventable hospitalization		Disparity in health status
Primary care physicians		Frequent mental distress
		Frequent physical distress
		Infant mortality
		Premature deaths

**Figure 6:**
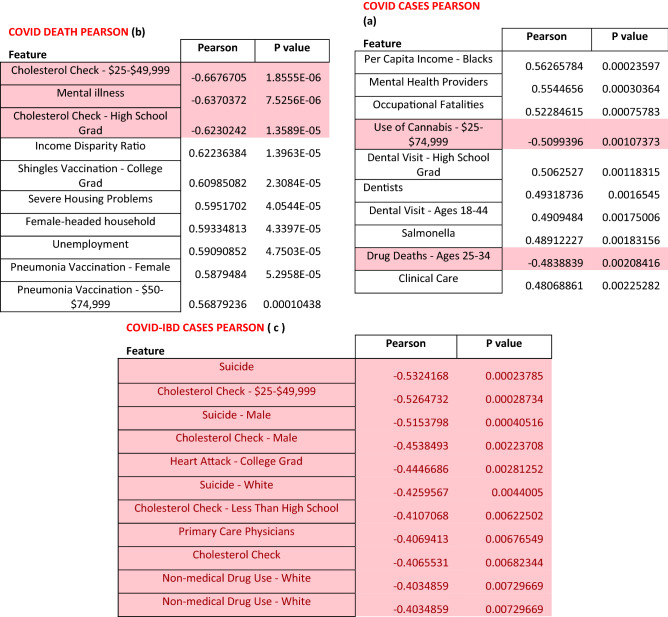
The health ranking criteria and their sub-categories for US states. Pearson correlation between (**a**) overall COVID cases and health ranking, (**b**) overall covid deaths and health rankings and (**c**) COVID-IBD cases and health rankings.

Although our initial hypothesis was that the healthy states would report few COVID-19 cases in IBD patients, we observed that the correlations with healthiness were negative for over 70% of the criteria. We provide the correlation and p values for the complete range of criteria reported in Fig. 6 (refer Supplementary Tables S4, S5 for full list). We tabulated the top 10 criteria (and correlations corresponding to the p values) in the increasing order of p values. Figure 6a–c show the Pearson correlation coefficients between (a) overall COVID cases and health ranking, (b) overall covid deaths and health rankings and (c) COVID-IBD cases and health rankings, with the negative correlations marked red. Across nearly all health indices, the healthy states report a higher number of COVID-19 cases in IBD patients. While this suggests that healthy states have more COVID-19/IBD cases, we believe that this may also imply an underreporting from the US states ranking low on the American health index rankings.

## Discussion

In this work, we carried out a comprehensive analysis of the clinical factors affecting the COVID-19 outcomes of IBD patients using machine learning methods. Current studies are severely hindered by the lack of patient samples. Thus, we employed a random sampling approach to generate a large COVID-19/IBD clinical dataset from published summary statistics. To verify the validity of these generated samples, we demonstrate that the relative rank of the odds ratio of the logistic regression analysis on the generated dataset largely aligns with that of the real COVID-19/IBD dataset. Our random sampling approach employs Bayesian statistics and a multinomial distribution making it highly generalizable with regard to the number of observable features.

The results from this work are significant in several ways. First, we showed that the sampling method we designed can preserve the summary statistics of the original data for the patient features, namely gender, age, medication usage, number of comorbidities, smoking status, IBD subtype, and IBD severity. This indicates that our methodology is robust and could be used more generally in cases where individual patient data is limited or unknown but summary statistics are available. Second, we determined key primary and secondary IBD patient features related to COVID-19 outcomes and evaluated their effect on COVID-19 mortality and their potential utility in predicting outcomes. Using several methods, such as random forest classifier, multiple linear regression and correlation analysis, we established, in keeping with almost all existing findings, that *age*, *medication usage* and *the number of comorbidities* is the primary features, while *gender* (male), *smoking* and *IBD severity* are secondary features affecting COVID-19 outcomes in IBD patients. These findings are consistent with existing studies on 79 and 232 patients with IBD with COVID-19 in Italy and the United States that show that old age and comorbidities pose a far greater risk of negative COVID-19 outcome than other factors^14,43^. Finally, our correlation studies reveal the non-uniformity in the reporting of COVID-IBD cases. Specifically, there seems to be reduced reporting from US states with poor health rankings, suggesting that more rigorous data collection is necessary before the repository can be used to derive demographic inferences.

We specifically note that to date, the relationship between IBD patients that smoke and COVID-19 outcomes have been unclear. Our principal components analysis suggests that male patients with ulcerative colitis and that smoke face high risks of mortality, especially elderly patients with multiple preexisting conditions. Existing studies suggest that smoking affects the pulmonary immune function, increasing the risk of contracting infectious diseases, and excessive smoking has been linked with the progression of COVID-19^44,45^. Our findings further suggest that the male IBD patients, in particular, are more likely to die due to COVID, lending credence to prior clinical studies that have shown that females are less susceptible to viral infections and reduced cytokine production, and that female patients have higher macrophage and neutrophil activity and antibody production and response^46^.

Of keen interest in IBD is whether any medications exacerbate the infection rate and/or severity of COVD-19. Our analysis shows that having UC and using 5-ASAs (sulfasalazine/mesalamine) are linked with higher COVID-19 deaths. In the early stages of the pandemic, there was a documented case of an 80-year-old female with a 3-year history of UC, in maintenance with mesalamine, who had a fever and bloody diarrhoea. She was diagnosed with COVID-19 pneumonia and passed away after 14 days of hospitalization^47^. As previously mentioned, there is considerable uncertainty among gastroenterologists and patient support groups about the effects of IBD medications on COVID-19 susceptibility and progression, and whether treatment guidelines need to be modified in the COVID era^15^. The present work supports reports that suggested 5-ASA usage was associated with adverse clinical outcomes such as hospitalization or death^39,48^. These findings on primary (age, medication usage, and the number of comorbidities) and secondary features (IBD severity, smoking status, gender and IBD subtype) increase our understanding of vulnerable IBD patient groups. Our findings suggest that gastroenterologists should weigh the risks and benefits before recommending 5-ASA to elderly patients with multiple comorbidities.

Our supervised machine learning classifiers using the three most discriminatory features (i.e., age, number of comorbidities and medication usage) show approximately 70% accuracy in predicting outcome, outperforming the accuracy for the complete feature set in a majority of the classification approaches. This suggests that these discriminatory features can be reasonably applied to prognosticate the risk associated with each IBD patient. Lastly, we demonstrated that the present COVID-19/IBD data repository appears to have fewer cases reported from unhealthy US states. This suggests that the current repository may not be useful for deriving accurate demographic information. Therefore, our proposed random sampling approach may be utilized in the future to generate a large-scale synthetic COVID-19/IBD data repository while taking into consideration the innate bias of data reporting.

## Supplementary Information

Supplementary Information.
