# Supplementary material for: A machine learning approach identifies 5-ASA and ulcerative colitis as being linked with higher COVID-19 mortality in patients with IBD
Source: Sci Rep. 2021 Aug 13;11:16522. doi: 10.1038/s41598-021-95919-2 (PMC8363640; doi:10.1038/s41598-021-95919-2)
Supplement: Supplementary file 1 — Supplementary Information. [file 41598_2021_95919_MOESM1_ESM.docx]

**A Machine Learning approach identifies 5-ASA and Ulcerative Colitis as being linked with higher COVID-19 mortality in patients with IBD**

# Satyaki Roy *·* Shehzad Sheikh *·* Terrence Furey

Received: date / Accepted: date

# Supplementary Materials

# Validation of data generation approach

We validate the efficacy of the proposed data generation approach by comparing the results of the multivariable logistic regression applied on the actual SECURE-IBD dataset by Brenner et al. [15] to identify factors associated with severe COVID-19. In order to compare the odd ratios of logistic regression on the generated dataset against those on the original data, we do the following:

- Combine (i) medication Budesonide and Oral/parenteral steroids into a single category called Systemic corticosteroid, (ii) medication Anti-TNF without6MP/AZA/MTX and Anti-TNF + 6MP/AZA/MTX into a category Anti-TNF and (iii) Comorbidity 2 and 3+ into a categoryComorbidity≥2.
- Rank the odds ratio in the logistic regression from [15] and the generated dataset, and compare their relative ordering using Kendall’s tau (discussed in Sec. 2.3).


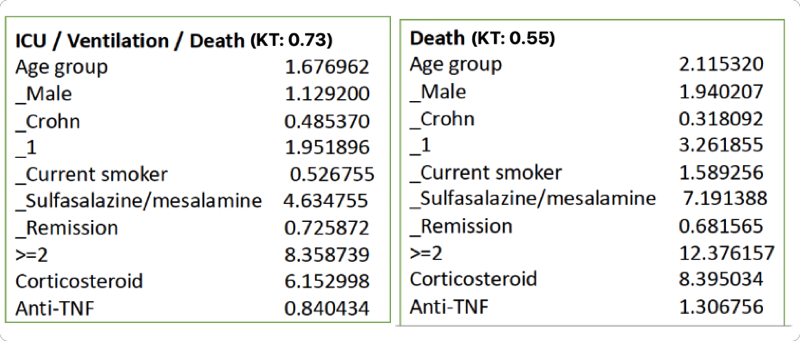


**Supplementary Figure S1.** List of input features ranked in the decreasing order of odds ratio on the generated dataset for outcomes (a) ICU / ventilation / death and (b) death.

Considering two outcome scenarios, namely (a) ICU / Ventilation / Death and (b) death, the list of input features ranked in the decreasing order of odds ratio on the generated dataset has a Kendall’s tau score of (a) 0.73 and (b) 0.55, respectively (as illustrated in Supplementary Figure S1). (The logistic regression results are in the supplementary materials.) Moreover, the relative rank within the odds ratio of the comorbidity categories 1 ≥ 2 and medication categories Systemic corticosteroid, Anti-TNF and Sulfasalazine in the original study is preserved in our logistic regression study based on generated dataset.

**2. Multiple regression analysis on features against death**

Given the high potential of mutual information between age and comorbidity, we studied two feature combinations with either age or comorbidity, i.e., (1) medication, condition, severity and age and (2) medication, condition, severity and comorbidity, against outcomes (outpatients, hospitalized/recovered and hospitalized/deceased). Supplementary Tables S1 and S2 show the corresponding multiple linear regression analysis, where age and comorbidity have the highest coefficients in their respective groups, followed by severity and IBD condition.

| **OLS Regression Results** |  |  |  |  |  |  |
| --- | --- | --- | --- | --- | --- | --- |
| **Dep. Variable:** | Status | R-squared: | 0.172 |  |  |  |
| **Model:** | OLS | Adj. R-squared: | 0.172 |  |  |  |
| **Method:** | Least Squares | F-statistic: | 1037 |  |  |  |
| **Covariance Type:** | Non-robust |  |  |  |  |  |
|  | Coef. | std err | t | P>\|t\| | [0.025 | 0.975] |
| **const** | -0.0837 | 0.009 | -9.289 | 0.000 | -0.101 | -0.066 |
| **Age group** | 0.0957 | 0.002 | 55.266 | 0.000 | 0.092 | 0.099 |
| **Medication** | -0.0228 | 0.001 | -21.679 | 0.000 | -0.025 | -0.021 |
| **Condition** | 0.0867 | 0.006 | 13.913 | 0.000 | 0.074 | 0.099 |
| **Severity** | 0.0400 | 0.003 | 11.979 | 0.000 | 0.033 | 0.046 |

**Supplementary Table S1.** Multiple linear regression between medication, condition, age and severity and COVID-IBD outcome

This explains why age and comorbidity exhibit such a high feature importance. The other significant features affecting outcomes are IBD condition and disease severity. It is imperative to note that these findings are all in sync with the rank of the feature importance obtained by employing the extra-trees classification machine learning approach.

| **OLS Regression Results** |  |  |  |  |  |  |
| --- | --- | --- | --- | --- | --- | --- |
| **Dep. Variable:** | Status | R-squared: | 0.193 |  |  |  |
| **Model:** | OLS | Adj. R-squared: | 0.193 |  |  |  |
| **Method:** | Least Squares | F-statistic: | 1195 |  |  |  |
| **Covariance Type:** | Non-robust |  |  |  |  |  |
|  | Coef. | std err | t | P>\|t\| | [0.025 | 0.975] |
| **const** | 0.1543 | 0.007 | 23.468 | 0.000 | 0.141 | 0.167 |
| **Comorbidity** | 0.2284 | 0.004 | 60.493 | 0.000 | 0.221 | 0.236 |
| **Medication** | -0.0229 | 0.001 | -22.149 | 0.000 | -0.025 | -0.021 |
| **Condition** | 0.0838 | 0.006 | 13.615 | 0.000 | 0.072 | 0.096 |
| **Severity** | 0.0395 | 0.003 | 11.985 | 0.000 | 0.033 | 0.046 |

# Supplementary Table S2. Multiple linear regression between medication, condition, comorbidity and severity and COVID-IBD outcome

# 3. Mortality due to COVID in IBD patients

# Given the sets of patient IDs belonging to upper region (PC2 $\boldsymbol{\geq1.5)}$ and lower region (PC2 $\boldsymbol{<1.5)}$ be represented by $\boldsymbol{\Gamma}$ and $\boldsymbol{\gamma}$*,* respectively (see PCA plot in Supplementary Figure S2)*.* We perform *hypothesis testing* (*one sample t-testing)* of the following three scenarios. As per the t-testing approach, the greater the magnitude of t-value, the greater the evidence against the null hypothesis. Given any condition $\boldsymbol{c}$, $\boldsymbol{x= 1}_{\boldsymbol{c}}$ is 1 (or true if the condition is met, and 0 otherwise.

(1) *Mortality in upper and lower regions.* Given $V\_\Gamma=\left\{ \boldsymbol{1}_{c} \right|c:outcome(p)=death \forall p\in\Gamma\}$ and $V\_\gamma=\left\{ \boldsymbol{1}_{c} \right|c:outcome(p)=death \forall p\in\gamma\}$, we define hypothesis (1) $H_{0}^{\Gamma}:mean\left( V^{\Gamma} \right)=0$ and hypothesis (2) $H_{0}^{\gamma}:mean\left( V^{\gamma} \right)=0$. (If hypothesis 1 or 2 is true, it implies that there are no deaths in the upper and lower region shown in Figure S4.) For upper and lower we get a t-statistic 4.76 and 21.6, respectively, with p values $2.32 \times{10}^{-6}$ and $1.39 \times{10}^{-102}$, respectively. Therefore, there are deaths in both the regions.

(2) *Mortality given Crohn’s and UC in upper*. Given ${V^{Crohn}}_{\Gamma}=\left\{ \boldsymbol{1}_{c} \right|c:outcome\left( p \right)=death \forall p\in\Gamma and condition\left( p \right)=Crohn\}$ and ${V^{UC}}_{\Gamma}=\left\{ \boldsymbol{1}_{c} \right|c:outcome\left( p \right)=death \forall p\in\Gamma and condition\left( p \right)=UC\}$, we define hypothesis (1) $H_{0}^{\mathrm{Crohn}}:mean\left( V^{\mathrm{Crohn}} \right)=0$ and hypothesis (2) $H_{0}^{\mathrm{UC}}:mean\left( V^{\mathrm{UC}} \right)=0$. In the two hypotheses, we are calculating the number of mean deaths in the upper region due to Crohn’s and UC, respectively. For Crohn and UC, we get a t-statistic 2.00 and 4.76, respectively, with p values $0.045$ and $2.32 \times{10}^{-6}$, respectively.

(3) *Mortality given Crohn’s and UC in lower.* Given ${V^{Crohn}}_{\gamma}=\left\{ \boldsymbol{1}_{c} \right|c:outcome\left( p \right)=death \forall p\in\gamma and condition\left( p \right)=Crohn\}$ and ${V^{UC}}_{\gamma}=\left\{ \boldsymbol{1}_{c} \right|c:outcome\left( p \right)=death \forall p\in\gamma and condition\left( p \right)=UC\}$, we define $H_{0}^{\mathrm{Crohn}}:mean\left( V^{\mathrm{Crohn}} \right)=0$ and $H_{0}^{\mathrm{UC}}:mean\left( V^{\mathrm{UC}} \right)=0$.

Like in part (2), in the two hypotheses, we are calculating the number of mean deaths in the lower region due to Crohn’s and UC, respectively. For Crohn and UC, we get a t-statistic 12.16 and 21.6, respectively, with p values $7.99 \times{10}^{-34}$ and $1.39 \times{10}^{-102}$, respectively. In parts (2) and (3), there is a higher t-value of mortality in COVID among UC patients than in Crohn’s patients.

#
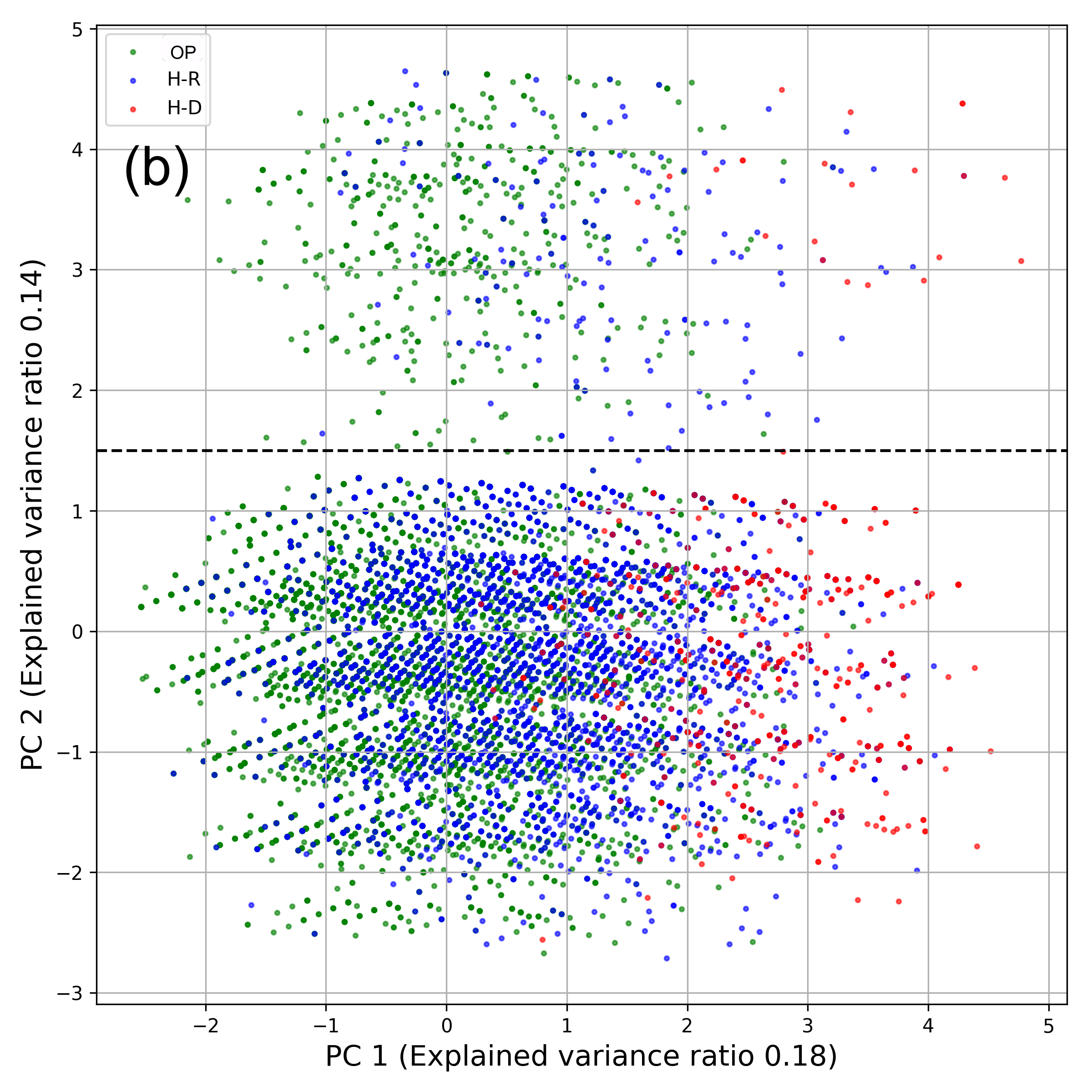


# Supplementary Figure S2. Identification of discriminatory features. (a) cosine similarity of medication and outcome vectors, (b) PCA on the 7 input features labeled by three patient outcomes

# 4. Accuracy of the supervised machine learning methods on different combination of feature sets

# We apply supervised machine learning support vector machine (SVM), stochastic gradient descent (SGD), nearest centroid (NC), decision tree classifier (DTC), nearest neighbor (NN) and naïve Bayes (NB) on different combinations of features sets, namely, (1) age, medication, comorbidity, (2) age, (3) medication and (4) comorbidity. Supplementary Table S3 shows that (age, medication and comorbidity) achieve the highest accuracy.

| **Approach** | **Age + Med + Com** | **Age** | **Medication** | **Comorbidity** |
| --- | --- | --- | --- | --- |
| SVM | 0.6515, 0.0076 | 0.3728, 0.0046 | 0.6018, 0.0069 | 0.4930, 0.0078 |
| SGD | 0.6011, 0.0080 | 0.3342, 0.0187 | 0.5841, 0.0099 | 0.4108, 0.0280 |
| NC | 0.5886, 0.0092 | 0.3728, 0.0046 | 0.6041, 0.0066 | 0.4803, 0.0072 |
| DTC | 0.6785, 0.0066 | 0.3728, 0.0046 | 0.6018, 0.0069 | 0.4974, 0.0054 |
| NN | 0.6468, 0.0109 | 0.3728, 0.0046 | 0.6031, 0.0056 | 0.4810, 0.0137 |
| NB | 0.6072, 0.0067 | 0.3728, 0.0046 | 0.6031, 0.0070 | 0.4850, 0.0066 |
| **Mean accuracy** | 0.6289 | 0.03663 | 0.5996 | 0.4745 |

**Supplementary Table S3.** The (accuracy, standard deviation) of the supervised machine learning approaches on input features (1) age, medication, comorbidity, (2) age, (3) medication and (4) comorbidity.

# 5. Reporting of IBD cases from the states of USA

We compare the overall COVID-19 infected and death numbers against ranked list of American health rankings to observe to get a positive correlation for (1) over 80% and (2) 60% features, respectively (Supplementary Tables S4 and S5). Note that the negative correlations are highlighted in red. Since it is highly unlikely that the healthier states have greater COVID-19 cases in IBD patients, we intuit that the high negative correlation between health and COVID-IBD rankings is a result of severe under-reporting on the part of the less health-conscious US states. Here we have the full-list of the correlation (for criteria satisfying p value $\leq0.05$) between (a) overall COVID cases and health ranking (b) overall covid deaths and health rankings and (c) COVID-IBD cases and health rankings.

**Supplementary Table S4.** The health ranking criteria and their sub-categories for US states – Pearson correlation between overall COVID cases and health ranking

| **Feature** | **Pearson** | **P value** |
| --- | --- | --- |
| Per Capita Income - Blacks | 0.562657836 | 0.000235973 |
| Mental Health Providers | 0.554465604 | 0.000303642 |
| Occupational Fatalities | 0.522846154 | 0.000757832 |
| Use of Cannabis - $25-$74,999 | -0.509939641 | 0.001073728 |
| Dental Visit - High School Grad | 0.506252699 | 0.001183152 |
| Dentists | 0.493187362 | 0.0016545 |
| Dental Visit - Ages 18-44 | 0.490948401 | 0.001750057 |
| Salmonella | 0.48912227 | 0.001831557 |
| Drug Deaths - Ages 25-34 | -0.483883905 | 0.002084155 |
| Clinical Care | 0.480688614 | 0.002252821 |
| Dental Visit - Less Than $25,000 | 0.478832738 | 0.002356183 |
| Uninsured | 0.476743858 | 0.002477471 |
| Drug Deaths - Male | -0.471818776 | 0.002785309 |
| Avoided Care Due to Cost - Ages 18-44 | 0.468380562 | 0.00301956 |
| Dental Visit - White | 0.461855422 | 0.003511766 |
| Physical Inactivity - Male | 0.458207228 | 0.003816314 |
| Drug Deaths | -0.45557605 | 0.004049982 |
| Exercise - Ages 45-64 | 0.444318467 | 0.005195701 |
| Dental Visit - College Grad | 0.443162498 | 0.005327849 |
| Primary Care Physicians | 0.441406946 | 0.005534095 |
| Drug Deaths - Ages 35-44 | -0.438865373 | 0.005844889 |
| Cardiovascular Diseases - Ages 65+ | 0.433406025 | 0.006563768 |
| Dental Visit - Male | 0.428983969 | 0.007200638 |
| Avoided Care Due to Cost - Male | 0.428287431 | 0.007305643 |
| Dental Visit - $25-$49,999 | 0.425153353 | 0.007794545 |
| Dental Visit - Some College | 0.422127059 | 0.008292999 |
| Use of Cannabis | -0.414979009 | 0.009579858 |
| Exercise - Male | 0.414939788 | 0.009587363 |
| Exercise | 0.413662243 | 0.009834613 |
| Dental Visit | 0.412570814 | 0.010050131 |
| Asthma - Some College | -0.410192213 | 0.010533805 |
| Per Capita Income | 0.409712909 | 0.01063363 |
| Asthma - $25-$49,999 | -0.407717766 | 0.011057862 |
| Dental Visit - Female | 0.404697563 | 0.011727402 |
| Severe Housing Problems - White | -0.404693968 | 0.011728219 |
| High Health Status - Ages 65+ | 0.401839869 | 0.012392303 |
| Avoided Care Due to Cost | 0.401222869 | 0.01253998 |
| Obesity | 0.400001262 | 0.012836773 |
| Use of Cannabis - White | -0.395989408 | 0.013853664 |
| Dental Visit - Ages 45-64 | 0.395458388 | 0.013993231 |
| Dental Visit - Ages 65+ | 0.395286521 | 0.014038656 |
| Drug Deaths - Ages 45-54 | -0.394406032 | 0.014273332 |
| Drug Deaths - White | -0.389949414 | 0.015512592 |
| Dedicated HC Provider - 18-44 | 0.389647912 | 0.015599607 |
| Avoided Care Due to Cost - Less Than $25,000 | 0.3896455 | 0.015600305 |
| Dedicated HC Provider - White | 0.389621805 | 0.015607161 |
| Obesity - College Grad | 0.388239587 | 0.016011519 |
| Asthma - Female | -0.385433491 | 0.016859587 |
| Exercise - Ages 18-44 | 0.384388996 | 0.017184751 |
| Dedicated HC Provider - $50-$74,999 | 0.383891008 | 0.017341624 |
| HPV Immunization Males | 0.383723771 | 0.017394575 |
| Infectious Disease | 0.379895051 | 0.018644476 |
| Dental Visit - $75,000 or More | 0.37972269 | 0.018702468 |
| Obesity - Ages 65+ | 0.378598155 | 0.019084548 |
| Obesity - Less Than $25,000 | 0.374272668 | 0.020615643 |
| All Determinants | 0.373666736 | 0.020838093 |
| Avoided Care Due to Cost - White | 0.370416937 | 0.022065619 |
| Obesity - Ages 18-44 | 0.370360288 | 0.022087539 |
| High Blood Pressure - College Grad | 0.370260199 | 0.022126313 |
| Drive Alone to Work | 0.369943379 | 0.02224942 |
| Exercise - White | 0.369106432 | 0.022577379 |
| Obesity - Male | 0.367858831 | 0.023073713 |
| Drug Deaths - Female | -0.366848983 | 0.023482074 |
| Asthma | -0.365303897 | 0.024118485 |
| Avoided Care Due to Cost - High School Grad | 0.365149979 | 0.024182659 |
| Seat Belt Use - College Grad | 0.36504622 | 0.024226 |
| Avoided Care Due to Cost - Less Than High School | 0.361772796 | 0.025626804 |
| Dedicated HC Provider - Male | 0.361730076 | 0.025645519 |
| Dedicated HC Provider - Some College | 0.360766291 | 0.026070764 |
| Exercise - Ages 65+ | 0.35941465 | 0.026676957 |
| Asthma - $75,000 or More | -0.358619585 | 0.027038944 |
| Dedicated Health Care Provider | 0.358261524 | 0.027203287 |
| Pertussis | -0.35749486 | 0.027557947 |
| Obesity - Ages 45-64 | 0.356567412 | 0.02799208 |
| Physical Inactivity - Some College | 0.356160446 | 0.028184353 |
| Dedicated HC Provider - $75,000 or More | 0.353268023 | 0.029582507 |
| Dedicated HC Provider - College Grad | 0.352684856 | 0.029871206 |
| Heart Disease | 0.351862859 | 0.030282073 |
| Obesity - Female | 0.351849376 | 0.03028885 |
| Population under 18 years | 0.351816707 | 0.030305278 |
| Physical Inactivity - Ages 45-64 | 0.351671274 | 0.030378499 |
| Exercise - College Grad | 0.351181438 | 0.030626188 |
| Median Household Income | 0.350341537 | 0.031054752 |
| Heart Disease - Ages 45-64 | 0.350281533 | 0.031085557 |
| Cardiovascular Diseases - Less Than $25,000 | 0.348611759 | 0.031952912 |
| Stroke - Less Than $25,000 | 0.348021254 | 0.03226436 |
| Heart Attack - Ages 65+ | 0.347149096 | 0.032728911 |
| Asthma - Ages 18-44 | -0.345944126 | 0.033379741 |
| Dedicated HC Provider - 45-64 | 0.345762569 | 0.033478717 |
| Stroke - Ages 65+ | 0.343987629 | 0.034459041 |
| Physical Inactivity - Ages 65+ | 0.343175398 | 0.034915413 |
| Dedicated HC Provider - Female | 0.342226862 | 0.035454613 |
| Exercise - $75,000 or More | 0.341501172 | 0.035871711 |
| Avoided Care Due to Cost - Female | 0.341074488 | 0.036118817 |
| Physical Inactivity - $75,000 or More | 0.340484893 | 0.036462554 |
| Dependency - White | 0.34022372 | 0.03661567 |
| Dental Visit - $50-$74,999 | 0.338751329 | 0.037488719 |
| Per Capita Income - White | 0.338580894 | 0.037590863 |
| Obesity - White | 0.336154811 | 0.039069603 |
| HPV Immunization Males - Above Poverty | 0.334668622 | 0.039998587 |
| Drug Deaths - Ages 55-64 | -0.334034167 | 0.040400596 |
| Exercise - Female | 0.331593829 | 0.041977547 |
| Dedicated HC Provider - High School Grad | 0.32439197 | 0.046924138 |
| Physical Inactivity - White | 0.323504514 | 0.04756489 |
| Physical Inactivity | 0.322819189 | 0.048064505 |
| Limited Access to Healthy Food | 0.322341339 | 0.048415359 |
| Seat Belt Use - $75,000 or More | 0.322026475 | 0.048647666 |
| Avoided Care Due to Cost - Ages 45-64 | 0.321793654 | 0.048820018 |
| Heart Attack - Ages 45-64 | 0.321130071 | 0.049313945 |
| Overall | 0.321022067 | 0.049394715 |
| Heart Disease - Ages 65+ | 0.320919386 | 0.049471602 |
| Neighborhood Amenities | 0.320899613 | 0.049486418 |
| Cholesterol Check - White | 0.320831792 | 0.049537268 |
| Exercise - $25-$49,999 | 0.320513679 | 0.049776335 |

**Supplementary Table S5.** The health ranking criteria and their sub-categories for US states -- overall covid deaths and health rankings

| **Feature** | **Pearson** | **P value** |
| --- | --- | --- |
| Cholesterol Check - $25-$49,999 | -0.667670507 | 1.85554E-06 |
| Mental illness | -0.63703718 | 7.52557E-06 |
| Cholesterol Check - High School Grad | -0.62302424 | 1.35886E-05 |
| Income Disparity Ratio | 0.62236384 | 1.39625E-05 |
| Shingles Vaccination - College Grad | 0.609850819 | 2.30841E-05 |
| Severe Housing Problems | 0.595170199 | 4.05445E-05 |
| Female-headed household | 0.593348129 | 4.33972E-05 |
| Unemployment | 0.590908522 | 4.7503E-05 |
| Pneumonia Vaccination - Female | 0.587948398 | 5.29582E-05 |
| Pneumonia Vaccination - $50-$74,999 | 0.56879236 | 0.000104384 |
| Pneumonia Vaccination | 0.562053788 | 0.000131229 |
| Shingles Vaccination - $50-$74,999 | 0.553852947 | 0.000172243 |
| Cholesterol Check - Less Than $25,000 | -0.553309589 | 0.000175331 |
| Unemployed | 0.553196592 | 0.00017598 |
| Suicide | -0.550360453 | 0.000192982 |
| Suicide - Male | -0.549284399 | 0.000199811 |
| Cholesterol Check - College Grad | -0.546652932 | 0.000217438 |
| Cholesterol Check - Ages 45-64 | -0.546223993 | 0.000220441 |
| Insufficient Sleep - College Grad | 0.52517077 | 0.000422699 |
| Substance misuse | -0.520212778 | 0.000489745 |
| Immunizations - Adults | 0.517581171 | 0.000529069 |
| Unemployment - White | 0.515223567 | 0.000566674 |
| Shingles Vaccination - Some College | 0.514087945 | 0.000585627 |
| Dependency - Hispanic | -0.510213497 | 0.000654626 |
| Pneumonia Vaccination - College Grad | 0.507019382 | 0.000716876 |
| Cholesterol Check - White | -0.504781029 | 0.000763592 |
| Shingles Vaccination - $25-$49,999 | 0.503735984 | 0.000786318 |
| Flu Vaccination - College Grad | 0.501072768 | 0.000846983 |
| Physical Inactivity - Ages 18-44 | 0.500997413 | 0.000848758 |
| Shingles Vaccination | 0.498943657 | 0.000898443 |
| Meningococcal Immunization | -0.496864082 | 0.000951375 |
| Shingles Vaccination - Male | 0.496660288 | 0.000956708 |
| Cholesterol Check | -0.496579579 | 0.000958828 |
| Pneumonia Vaccination - $25-$49,999 | 0.49005822 | 0.001144534 |
| Shingles Vaccination - $75,000 or More | 0.489835502 | 0.001151405 |
| Concentrated Disadvantage | 0.486119162 | 0.001271578 |
| Cholesterol Check - Ages 18-44 | -0.479274266 | 0.001522307 |
| Cholesterol Check - Female | -0.477309271 | 0.001601933 |
| Shingles Vaccination - Female | 0.474580324 | 0.001718618 |
| Meningococcal Immunization - White | -0.472936648 | 0.001792466 |
| Preventable Hospitalizations - Hispanic | 0.470622654 | 0.001901175 |
| Cancer - Less Than $25,000 | -0.468995384 | 0.001981065 |
| Meningococcal Immunization - Above Poverty | -0.465568789 | 0.002159033 |
| Shingles Vaccination - High School Grad | 0.458199043 | 0.002590182 |
| Physical Inactivity - $25-$49,999 | 0.4529256 | 0.002943435 |
| Cholesterol Check - $50-$74,999 | -0.452515281 | 0.002972613 |
| Injury Deaths - Female | -0.452484643 | 0.002974801 |
| Cholesterol Check - Male | -0.452369326 | 0.002983052 |
| Physical Inactivity - $50-$74,999 | 0.443780205 | 0.003656861 |
| Shingles Vaccination - White | 0.443369519 | 0.003692167 |
| Pneumonia Vaccination - Male | 0.440644053 | 0.003934106 |
| Insufficient Sleep - $75,000 or More | 0.439837857 | 0.004008272 |
| Low Birthweight - College Graduate | 0.439061972 | 0.004080798 |
| Severe Housing Problems - Hispanic | 0.436033036 | 0.004374992 |
| Insufficient Sleep - $25-$49,999 | 0.433438054 | 0.004641554 |
| Underemployment Rate | 0.432090577 | 0.00478546 |
| Insufficient Sleep - Ages 65+ | 0.430605723 | 0.004948514 |
| Cholesterol Check - Less Than High School | -0.429971348 | 0.005019634 |
| Unemployment Rate, Annual | 0.424996394 | 0.005608826 |
| Pneumonia Vaccination - Less Than High School | 0.424181845 | 0.005710793 |
| Pneumonia Vaccination - Some College | 0.422392193 | 0.005940483 |
| Cholesterol Check - Some College | -0.416980811 | 0.006684308 |
| Insufficient Sleep - Male | 0.416423749 | 0.006765258 |
| Pertussis | -0.412142534 | 0.007416128 |
| Substandard Housing | -0.411200248 | 0.007566413 |
| Insufficient Sleep | 0.404428634 | 0.00872562 |
| Smoking - Less Than $25,000 | -0.400387355 | 0.009487606 |
| Colorectal Cancer Screening - Less Than High School | -0.398246675 | 0.00991384 |
| Physical Inactivity - Female | 0.39667118 | 0.010237913 |
| Cholesterol Check - Ages 65+ | -0.392282753 | 0.011188754 |
| Suicide - White | -0.388589263 | 0.01204645 |
| Drug Deaths - White | 0.387739258 | 0.012251573 |
| Education - Less Than HS | 0.383750637 | 0.013254267 |
| Drug Deaths - Male | 0.379747669 | 0.014329793 |
| Tdap Immunization | -0.37897498 | 0.014545695 |
| Flu Vaccination - Female | 0.378558887 | 0.014663096 |
| Seat Belt Use - White | -0.374835448 | 0.015749844 |
| Seat Belt Use - Ages 45 to 64 | -0.374832979 | 0.015750587 |
| Pneumonia Vaccination - White | 0.373068551 | 0.016288891 |
| Depression - College Grad | -0.371526248 | 0.016772069 |
| Shingles Vaccination - Less Than $25,000 | 0.368675726 | 0.01769693 |
| Insufficient Sleep - Female | 0.36834257 | 0.017807774 |
| Seat Belt Use - $25-$49,999 | -0.364862482 | 0.019000975 |
| High-risk HIV Behaviors - Ages 45-64 | 0.364807649 | 0.019020299 |
| Depression - Some College | -0.364302607 | 0.019199069 |
| Injury Deaths - Ages 65-74 | -0.36421647 | 0.019229699 |
| Flu Vaccination - $50-$74,999 | 0.363437619 | 0.019508525 |
| Smoking - Less Than High School | -0.361768074 | 0.020117662 |
| Pneumonia Vaccination - Less Than $25,000 | 0.360331683 | 0.020654418 |
| Flu Vaccination - 18-44 | 0.359609347 | 0.020928844 |
| High Cholesterol - $75,000 or More | 0.35819677 | 0.021474328 |
| Cholesterol Check - $75,000 or More | -0.355850625 | 0.022406585 |
| Seat Belt Use - High School Grad | -0.352628101 | 0.023741969 |
| Seat Belt Use - Male | -0.352237471 | 0.023908259 |
| Seat Belt Use - Ages 65+ | -0.34966234 | 0.025028937 |
| Depression - $75,000 or More | -0.349039845 | 0.025306302 |
| Seat Belt Use - Some College | -0.347517566 | 0.025995366 |
| Diabetes - Less Than High School | 0.346474 | 0.026476684 |
| Pneumonia Vaccination - $75,000 or More | 0.344559594 | 0.027378888 |
| Immunizations - Adolescents | -0.341650805 | 0.028798379 |
| Low Birthweight - Mother Ages 25-29 | 0.3392902 | 0.029994623 |
| Pneumonia Vaccination - High School Grad | 0.338822546 | 0.030236411 |
| Frequent Mental Distress - Ages 65+ | 0.337648263 | 0.030850642 |
| Depression - Female | -0.337098601 | 0.031141669 |
| Drug Deaths - Ages 25-34 | 0.336285643 | 0.031576249 |
| High-risk HIV Behaviors - Male | 0.33599483 | 0.031732916 |
| Insufficient Sleep - Some College | 0.335380776 | 0.032065825 |
| Per Capita Income - Hispanics | -0.334092477 | 0.032773623 |
| Low Birthweight - Mother Ages 15-19 | 0.333061747 | 0.033349123 |
| Seat Belt Use - $75,000 or More | -0.332174431 | 0.033851178 |
| Cancer - $50-$74,999 | 0.329620588 | 0.035330928 |
| Receiving public assistance | 0.327666718 | 0.0364985 |
| Seat Belt Use | -0.327497019 | 0.03660138 |
| Depression - Less Than $25,000 | -0.326902169 | 0.036963884 |
| Seat Belt Use - $50-$74,999 | -0.325612174 | 0.037760109 |
| Insufficient Sleep - Ages 45-64 | 0.325033068 | 0.038122082 |
| Chlamydia | 0.322002829 | 0.040062655 |
| Depression - Ages 18-44 | -0.318244238 | 0.042580739 |
| Multiple Chronic Conditions - $50-$74,999 | 0.31782687 | 0.042868116 |
| Avoided Care Due to Cost - Ages 65+ | 0.314537813 | 0.045188321 |
| Preventable Hospitalizations | 0.312667396 | 0.046552488 |
| Physical Inactivity | 0.312096536 | 0.046975407 |
| Chronic Kidney Disease - Male | 0.311040483 | 0.047765965 |
| Diabetes - $50-$74,999 | 0.310319683 | 0.048311696 |
